# Improvement of genetic stability in lymphocytes from Fanconi anemia patients through the combined effect of α-lipoic acid and N-acetylcysteine

**DOI:** 10.1186/1750-1172-7-28

**Published:** 2012-05-16

**Authors:** Filipa Ponte, Rosa Sousa, Ana Paula Fernandes, Cristina Gonçalves, José Barbot, Félix Carvalho, Beatriz Porto

**Affiliations:** 1 Chemistry and Technology Network (REQUIMTE), Laboratory of Toxicology, Department of Biological Sciences, Faculty of Pharmacy, University of Porto, Porto, Portugal; 2 Cytogenetics Laboratory, Department of Microscopy, Institute of Biomedical Sciences Abel Salazar (ICBAS), Porto, Portugal; 3 Hemato-oncologic Unity, Hospital São João (HSJ), Porto, Portugal; 4 Service of Hematology of Hospital Center of Porto (CHP), Porto, Portugal; 5 Unity of Pediatric Hematology of CHP, Porto, Portugal

**Keywords:** Fanconi Anemia, Oxidative stress, Antioxidants, α-lipoic acid, N-acetylcysteine, Chromosome instability, Bone marrow failure, Cancer susceptibility

## Abstract

Fanconi Anemia (FA) is a rare genetic disorder, characterized by progressive bone marrow failure and increased predisposition to cancer. Despite being highly heterogeneous, all FA patients are hypersensitive to alkylating agents, in particular to 1,2:3,4-diepoxybutane (DEB), and to oxidative damage. Recent studies point to defective mitochondria in FA cells, which is closely related with increased production of reactive oxygen species (ROS) and concomitant depletion of antioxidant defenses, of which glutathione is a well-known biomarker.

The objective of the present work is to evaluate the putative protective effect of α-lipoic acid (α-LA), a mitochondrial protective agent, and N-acetylcysteine (NAC), a direct antioxidant and a known precursor for glutathione synthesis, in spontaneous and DEB-induced chromosome instability (CI) in lymphocyte cultures from FA patients.

For that purpose, lymphocyte cultures from 15 FA patients and 24 healthy controls were pre-treated with 20 μM α-LA, 500 μM NAC and α-LA plus NAC at the same concentrations, and some of them were exposed to DEB (0.05 μg/ml). A hundred metaphases per treatment were scored to estimate the relative frequency of spontaneous and DEB-induced chromosome breakage.

The obtained results revealed that a cocktail of α-LA and NAC can drastically improve the genetic stability in FA lymphocytes *in vitro,* decreasing CI by 60% and 80% in cultures from FA patients and FA mosaic/chimera patients, respectively. These results suggest that the studied cocktail can be used as a prophylactic approach to delay progressive clinical symptoms in FA patients caused by CI, which can culminate in the delay of the progressive bone marrow failure and early cancer development.

## Introduction

Fanconi Anemia (FA) is a rare genetic disorder characterized by chromosome instability (CI), several congenital malformations, progressive bone marrow failure, and increased predisposition to cancer, particularly acute myelogenous leukemia [1]. Genetically, FA is a highly heterogeneous disease with 15 genes so far characterized and cloned (FA-A, B, C, D1, D2, E, F, G, I, J, L, M, N, O, P) [2] and that appears to influence genomic stability. The clinical manifestations and the onset of disease are also extremely variable. However, all FA patients have, in common, unique markers that characterize the disease: hypersensitivity to the clastogenic effect of DNA cross-linking agents, in particular to diepoxybutane (DEB), [3] and hypersensitivity to oxidative damage [4].

DEB-induced cytotoxicity, despite hitherto not completely understood, has been related to oxidative damage and to glutathione (GSH) depletion [5-7]. The activation of the mitochondrial apoptotic pathway in the event of DEB-induced oxidative stress (OS) was more recently demonstrated [8]. Importantly, DEB-induced DNA-DNA and DNA-protein cross-links [9] are characteristic features of ROS-mediated damage. In accordance to the involvement of mitochondria and OS in the mechanisms of DEB toxicity, we have recently demonstrated that acute exposure to DEB both causes glutathione (GSH) depletion and loss of ATP, followed by cell death in lymphocytes collected from healthy humans [10].

The role of oxygen and oxidative damage in FA cells was described for the first time by Nordenson [11] followed by Joenje et al [12]. Two years later, Joenje and Oostra [13] established a direct correlation between high tensions of oxygen and increased damage in FA lymphocytes. A direct association of OS with the primary genetic defect in FA was suggested through the interaction of the FANCC protein with NADPH cytochrome P450 reductase [14] and glutathione S-transferase [15], two enzymes involved in the detoxification of reactive intermediates. At least FANCC, FANCG and FANCA have been shown to be associated with redox-related imbalance and to a unique functional and structural sensitivity to OS [16] and, altogether, these proteins are also related with mitochondrial dysfunction [14-18]. Recently, Mukhopadhyay et al [18] reported that FANCG protein physically interacts with the mitochondrial peroxidase peroxiredoxin 3 (PRDX3), FA-G cells display distorted structures, and FA-A, -C and -G have PRDX3 cleavage and decreased peroxidase activity.

Mitochondrial dysfunction ultimately leads to oxidative modifications of DNA, proteins and lipids. It is recognized that FA cells show abnormal accumulation of 8-hydroxy-2’-deoxyguanosine (8-OHdG), a sub-product of oxidative DNA damage, and also a mutagenic substance by itself [19]. However, Castillo et al [20] concluded that the FA pathway is redundant for the repair of oxidative DNA damage (7,8-dihydro-8-hydroxyguanine, 8-oxoG). Therefore, the accumulation of 8-oxoG in FA cells reflects sustained ROS overproduction rather than defective processing of oxidized bases. These evidences, altogether, point towards a pro-oxidant state associated with dysfunctional mitochondria, in FA cells (reviewed by Pagano et al) [4].

Some studies have been performed testing the use of antioxidants for decreasing chromosome damage in FA cells. Dallapiccola et al [21] obtained a partial correction of CI in FA lymphocytes treated with the antioxidants sodium ascorbate, L-cysteine, 2-mercaptoethanol, α-mercaptopropionylglycine, and GSH. Later, the same group [22] extended this study to desferoxamine, also with partial correction of CI. Pincheira et al [23] demonstrated that Vitamin E decreases the frequency of chromosomal damage and the duration of G2 in FA lymphocytes. Some *in vivo* studies were also performed in animal models. Zhang et al [24] showed that dietary supplements with antioxidants can delay the age of onset of epithelial tumors in FA mouse models. More recently, this group [25] also demonstrated that the antioxidant resveratrol maintains Fancd2(−/−) KSL cells in quiescence, improves the marrow microenvironment, partially corrects the abnormal cell cycle status, and significantly improves the spleen colony-forming capacity of Fancd2(−/−) bone marrow cells. In spite of the promising results from previous studies, no therapy using antioxidants, megavitamins, or micronutrients has been shown to be effective in treatment of FA, using evidence-based criteria. Thus, further studies with new protective agents are required for the application of drugs with high effectiveness.

Among a number of potential candidate molecules with antioxidant properties, some can be identified as “mitochondrial nutrients” and substances that normalize GSH balance. α-Lipoic acid (α-LA) is a natural and endogenous compound that occurs in mitochondria and is an essential cofactor for many mitochondrially localized enzyme complexes. Furthermore, α-LA features a cyclic disulfide moiety which exists in redox equilibrium with its reduced dithiol form, dihydrolipoic acid. Dihydrolipoic acid acts in GSH replenishment and is a potent antioxidant in a redox-cycling environment, by reducing vitamin C, vitamin E and coenzyme Q_10_[26,27]. Previous studies also proved that α-LA can be a protective agent against clastogenic [28] and genotoxic agents [29]. N-acetylcysteine (NAC), the acetylated variant of the amino acid L-cysteine, is an excellent source of sulfhydryl groups, and is converted in the body into metabolites capable of stimulating GSH synthesis, promoting detoxification, and acting directly as free radical scavengers [30]. Recently, a study from Yadavilli et al [8] showed that NAC has the ability to inhibit ROS production and apoptosis induced by DEB in human lymphoblasts. Importantly, there are some clinical studies referring the benefit of the combined use of α-LA and NAC by ameliorating the hematological response in advanced cancer patients [31] and in the event of muscle-damaging exercise [32].

In a previous study [10], we showed that α-LA and NAC have a partial protective effect on acute DEB-induced toxicity to normal human lymphocytes, though the effect of these compounds in CI of lymphocytes has never been tested before. Thus, the aim of the present work was to evaluate the putative protective effect of α-LA and NAC, in spontaneous and DEB induced CI lymphocytes cultures from FA patients.

## Methods

### Subjects

This study includes 15 FA patients, referred by several Hospitals, and already diagnosed with certainty on the basis of DEB-induced cytogenetic tests, and 24 healthy donors (HD), recruited among healthy male and female donors, aged 20–40 years. The study was approved by the Ethical Committe of Hospital Geral de Santo António, CHP, Porto. All procedures were done with the written informed consent of the participants.

### Cells and cell cultures

From each HD participant, 10 ml of venous blood was collected by antecubital venipuncture, into vacuum tubes with lithium heparin. From FA patients, 3 ml to 6 ml of venous blood were collected, depending on the age and healthy conditions of the patients, into vacuum tubes with lithium heparin.

Whole blood (0.5 ml) was cultured in RPMI 1640 (Sigma) complete medium supplemented with 15% fetal calf serum (GIBCO), antibiotics (10,000 units/ml of penicillin and 10,000 μg/ml of streptomycin) (GIBCO) and 29 mg/ml of L-glutamine (Sigma). Cultures were stimulated with 5 μg/ml of phytohemagglutinin (GIBCO) and placed in an incubator at 37°C with 5% CO_2_ atmosphere, for 72 h.

#### Exposure to DEB

**Figure 1:**
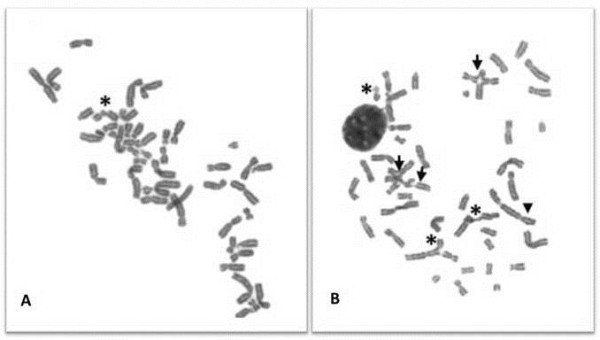
**CI pattern in metaphases from DEB-induced lymphocyte cultures from a healthy donor and FA patient.** Chromosomes were stained in a 4% Giemsa solution. The images were observed with an optical microscope (Olympus CX31) and captured with a digital camera (Nikon Sightds-smc), with the software DP20-5E microscope digital camera. (**A**) Selected metaphase from a HD lymphocyte culture exposed to 0.2 μg/ml of DEB for 48 h. One chromatid break can be seen (asterisk). (**B**) Selected metaphase from a FA patient lymphocyte culture exposed to 0.05 μg/ml of DEB for 48 h. High level of chromosome instability can be visualized, especially being important the tri and tetraradial figures (arrow) that are the hallmark for the diagnosis of FA. It can also be seen a dicentric chromosome (head arrow) and 3 chromatid breaks (asterisks).

DEB ((±)-1,2:3,4-diepoxybutane, [298-18-0], D-7019 Lot 34 H3683, Sigma), prepared in RPMI 1640, was added to lymphocyte cultures 24 h after their initiation, thus exposing cells to the chemical for 48 h. In lymphocyte cultures from FA patients, DEB was added at the concentration of 0.05 μg/ml (the same concentration used in the diagnostic test). As the lymphocytes from HD are not so sensitive to DEB at that concentration, and have variable responses, 3 higher concentrations were used to obtain a significant toxic response (see Figure 1 for a description of DEB-induced CI pattern in HD and FA patients). Therefore, a concentration of 0.1 μg/ml was selected in cultures from 5 individuals (HD1-5), a concentration of 0.2 μg/ml was selected in cultures from 11 individuals (HD6-11 and HD14-18) and a concentration of 0.4 μg/ml was selected in cultures from 8 individuals (HD12-13 and HD19-24).

Since DEB is a suspected carcinogen with unknown risk, appropriate precautions were taken. All culture procedures were handled using appropriate gloves and in a vertical laminar flow hood. As DEB is rapidly inactivated by concentrated HCl, all disposable culture bottles and pipettes were rinsed with HCl before being discarded.

### Antioxidant treatments

We used two antioxidants, α-LA 20 μM [33] (Sigma) and NAC 500 μM [10] (Sigma) at concentrations that proved to be effective in *in vitro* studies, preferably with lymphocytes. In addition, prospective studies were always performed to find the most efficient concentrations (data not shown). α-LA and NAC concentrations used in the present study are within the plasma concentrations found in previous human pharmacokinetic studies using these drugs [34,35]. α-LA was dissolved in 1% dimetilsulfoxide (DMSO) in RPMI 1640 and NAC was dissolved in RPMI 1640. α-LA was stored in the dark at room temperature. NAC was stored at 4°C. The two antioxidants were used from the same batch in all experiments.

#### Antioxidant treatments in DEB-exposed lymphocyte cultures from healthy donors

Two sets of experiments were done. In the first set, lymphocyte cultures were pre-treated with α-LA 20 μM 1.5 h before DEB exposure. In the second set of experiments lymphocyte cultures were pre-treated with NAC 500 μM, 1.5 h before DEB exposure. DMSO toxicity was found to be absent within our experimental conditions.

#### Antioxidant treatments in spontaneous and DEB-exposed lymphocyte cultures from FA patients

Three sets of experiments were done. In the first set, lymphocyte cultures were treated with α-LA 20 μM for 48 h. In DEB-induced lymphocyte cultures α-LA was added 1.5 h before DEB exposure. In the second, lymphocyte cultures were treated with NAC 500 μM for 48 h. In DEB-exposed lymphocyte cultures, NAC was added 1.5 h before DEB exposure. In the last set, both antioxidants were added simultaneously to lymphocyte cultures, at the same concentrations, for 48 h. In DEB-exposed lymphocyte cultures antioxidants were added 1.5 h before DEB exposure. DMSO toxicity was found to be absent within our experimental conditions.

### Cytogenetic analysis

After 3 days of culture, cells were harvested after 1 h of incubation with colcemid (GIBCO) (4 μg/ml), followed by hypotonic treatment with 75 mM KCl (Sigma) and fixed 3 times in a 1:3 iced solution of acetic acid (Merck): methanol (Merck). The resulting suspensions were dropped onto microscope slides and stained for 5 min in a 4% Giemsa solution (Merck) diluted in phosphate buffer saline solution (Sigma).

Analysis was performed on 50–100 metaphases from each experiment, by an independent scorer and in a blinded fashion. Only when the mitotic index (MI) was very low and the number of breaks was high, a minimum of 22 metaphases was counted. Each cell was scored for chromosome number and the number and type of structural abnormalities. Gaps (acromatic areas less than a chromatid in width) were excluded in the calculation of chromosome breakage frequencies, and rearrangements (triradials and quadriradials, dicentrics and ring chromosomes) were scored as two breaks.

### Mitotic index

From the same microscope stained slides, used for chromosome breakage analysis, a thousand cells were randomly scored, distinguishing between interphase nuclei and metaphase nuclei. MI was calculated as follows: number of metaphase nuclei /number of total nuclei.

### Statistical analysis

Graph results are expressed as mean ± SEM and table results are expressed as mean ± SD. Statistical comparison among groups was estimated using one-way ANOVA, followed by the Bonferroni post hoc test, and comparison between two groups was estimated using paired t-test, both with GraphPad Prism, version 5.0 software. P values lower than 0.05 were considered as statistically significant.

## Results

### Effect of α-lipoic acid and N-acetylcysteine on DEB-induced CI in lymphocyte cultures from healthy donors

As shown in Table 1, lymphocyte cultures from HD exposed to DEB and pre-treated with α-LA or NAC showed a reduction in the number of breaks per cell compared to those only exposed to DEB (*P* > 0.05 and *P* < 0.05, respectively). Noteworthy, two distinct groups can be considered concerning the protective effect: a group of responders and another one of non-responders. Among DEB-induced cultured lymphocytes pre-treated with α-LA 54% are responders whereas among those pre-treated with NAC, 82% are responders. However, the response with α-LA treatment is greater, while with NAC the potency is not only lower but also more variable. Globally, the percentage of Cl reduction in lymphocyte cultures pre-treated with α-LA was 25% and with NAC was 20%.

**Table 1:** Number of DEB-induced breaks per cell in cultured lymphocytes from healthy donors

Number of breaks per cell							
Healthy donors	Control group *(n)*	α-LA 20 μM *(n)*	% reduction	Healthy donors	Control group *(n)*	NAC 500 μM *(n)*	% reduction
HD1	0,09 *(100)*	0,09 *(100)*	0,00	HD14	0,20 *(41)*	0,14 *(50)*	30,00
HD2	0,09 *(100)*	0,06 *(100)*	33,33	HD15	0,14 *(50)*	0,12 *(50)*	14,29
HD3	0,07 *(100)*	0,07 *(100)*	0,00	HD16	0,12 *(50)*	0,04 *(50)*	66,67
HD4	0,08 *(100)*	0,03 *(100)*	62,50	HD17	0,10 *(50)*	0,12 *(50)*	0,00
HD5	0,04 *(100)*	0,01 *(100)*	75,00	HD18	0,12 *(50)*	0,08 *(50)*	33,33
HD6	0,44 *(100)*	0,12 *(100)*	72,73	HD19	0,22 *(50)*	0,16 *(50)*	27,27
HD7	0,12 *(100)*	0,11 *(100)*	8,33	HD20	0,32 *(50)*	0,14 *(50)*	56,25
HD8	0,08 *(100)*	0,09 *(100)*	0,00	HD21	0,14 *(50)*	0,10 *(50)*	28,57
HD9	0,09 *(100)*	0,10 *(100)*	0,00	HD22	1,14 *(50)*	1,00 *(50)*	12,28
HD10	0,10 *(100)*	0,11 *(100)*	0,00	HD23	0,18 *(51)*	0,12 *(50)*	33,33
HD11	0,18 *(50)*	0,08 *(100)*	55,56	HD24	0,13 *(100)*	0,20 *(50)*	0,00
HD12	0,19 *(53)*	0,22 *(100)*	0,00	_	_	_	_
HD13	0,15 *(100)*	0,06 *(100)*	60,00	_	_	_	_
Mean ± SD	0.132 ± 0.102	0.088 ± 0.051		Mean ± SD	0.255 ± 0.300	0.202 ± 0.268	

In control group no antioxidant treatment was done. The effect of the antioxidants was calculated by the percentage of reduction of the number of breaks per cell in α-LA and NAC treatments relatively to control group. ― Unavailable data; α-LA indicates α-lipoic acid; NAC indicates N-acetylcysteine; n indicates the number of cells analyzed. Paired t-test: Control group vs NAC 500 μM *P* < 0.05.

### Effect of α-lipoic acid, N-acetylcysteine and α-lipoic acid plus N-acetylcysteine on spontaneous CI in lymphocyte cultures from FA patients

**Figure 2:**
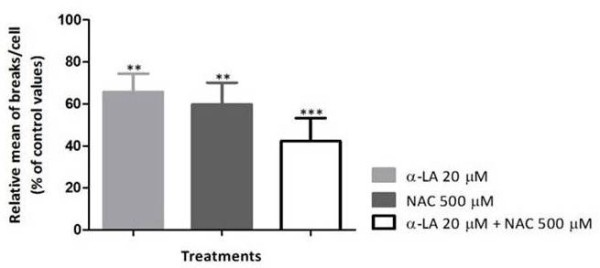
**Effect of α-LA, NAC and α-LA + NAC on chromosome breaks in cultured lymphocytes from FA patients.** Lymphocyte cultures were treated with 20 μM α-LA, 500 μM NAC, and both antioxidants simultaneously at the same concentrations. The results are presented as the relative mean of breaks per cell (percentage of control values). Comparison of the relative mean of breaks/cell was made between control group and each antioxidant treatment (*** *P* < 0.001 and **P < 0.01) and between treatments (*P* > 0.05). Results are representative of the mean of 14 experiments depicted from Table 2.

As shown in Table 2, lymphocyte cultures from FA patients treated with α-LA, NAC and α-LA plus NAC showed a significant reduction in the number of breaks per cell, compared to cultures without antioxidant treatment (*P* < 0.01, *P* < 0.01 and *P* < 0.01, respectively). Comparing α-LA *vs* NAC treatments, no difference (P > 0.05) was found in the reduction of the number of breaks per cell. However, comparing both isolated α-LA and NAC treatments with the combined α-LA plus NAC treatment, the reduction in the number of breaks per cell was highly significant (*P* < 0.05 and *P* < 0.01, respectively). Globally, the percentage of reduction in lymphocyte cultures treated with α-LA was 34% (*P* < 0.01), with NAC was 40% (*P* < 0.01) and with α-LA plus NAC was 58% (*P* < 0.001), as shown in Figure 2.

**Table 2:** Number of breaks per cell in cultured lymphocytes from FA patients

Mean number of breaks per cell							
Patients	Control group *(n)*	α-LA 20 μM *(n)*	% reduction	NAC 500 μM *(n)*	% reduction	α-LA 20 μM + NAC 500 μM *(n)*	% reduction
FA1	0.26 *(100)*	0.09 *(100)*	65.38	_	_	_	_
FA2	0.27 *(100)*	0.14 *(100)*	48.15	_	_	_	_
FA3	0.28 *(100)*	0.15 *(100)*	46.43	_	_	_	_
FA4	0.62 *(91)*	0.29 *(92)*	53.23	_	_	_	_
FA5	1.50 *(100)*	0.94 *(100)*	37.33	_	_	_	_
FA6	0.75 *(100)*	0.50 *(76)*	33.33	_	_	_	_
FA7	1.50 *(50)*	1.50 *(50)*	0.00	_	_	_	_
FA8	0.19 *(100)*	0.16 *(50)*	15.79	0.16 *(50)*	15.79	0.16 *(50)*	15.79
FA9	0.26 *(50)*	0.12 *(50)*	53.85	0.06 *(50)*	76.92	0.04 *(50)*	84.62
FA10	0.30 *(50)*	0.14 *(50)*	53.33	0.08 *(50)*	73.33	0.04 *(50)*	86.67
FA11	0.22 *(50)*	0.18 *(50)*	18.18	0.10 *(50)*	54.55	0.06 *(50)*	72.73
FA12	0.24 *(50)*	0.08 *(50)*	66.67	0.18 *(50)*	25.00	0.06 *(50)*	75.00
FA13	0.30 *(50)*	0.44 *(50)*	0.00	0.26 *(50)*	13.33	0.20 *(50)*	33.33
FA14	0.31 *(100)*	_	_	0.24 *(50)*	22.58	0.20 *(50)*	35.48
Mean ± SD	0.500 ± 0.452	0.364 ± 0.417		0.154 ± 0.078		0.109 ± 0.075	

In control group no antioxidant treatment was done. The effect of the antioxidants was calculated by the percentage of reduction of the number of breaks per cell in the α-LA, NAC and α-LA plus NAC treatments relatively to control group. ― Unavailable data. Abbreviations are explained in Table 1. Paired t-test: Control group vs α-LA 20 μM *P* < 0.01; Control group vs NAC 500 μM *P* < 0.01; Control group vs α-LA 20 μM + NAC 500 μM *P* < 0.01; α-LA 20 μM vs α-LA 20 μM + NAC 500 μM *P* < 0.05; NAC 500 μM vs α-LA 20 μM + NAC 500 μM *P* < 0.01.

Concerning the α-LA antioxidant effect two distinct groups were, once more, observed: responders and non-responders; in a total of 13 lymphocyte cultures, 85% were responders, and in two of them CI decreased to normal values (below 0.1). In NAC and α-LA plus NAC treated cultures only responders were observed. Besides, in two cultures treated with NAC and four treated with α-LA plus NAC a decrease in the number of breaks per cell to normal values was also observed (Table 2).

#### Effect of α-lipoic acid, N-acetylcysteine and α-lipoic acid plus N-acetylcysteine on spontaneous CI in lymphocyte cultures from a particular group of FA patients - mosaics and chimera

**Figure 3:**
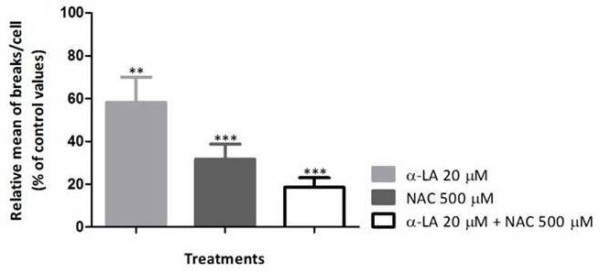
**Effect of α-LA, NAC and α-LA + NAC on chromosome breaks in cultured lymphocytes from FA mosaics/chimeras.** Lymphocyte cultures were treated with 20 μM α-LA, 500 μM NAC, and both antioxidants simultaneously at the same concentrations. The results are presented as the relative mean of breaks per cell (percentage of control values). Comparison of the relative mean of breaks/cell was made between control group and each antioxidant treatment (*** *P* < 0.001 and **P < 0.01) and between treatments (*P* > 0.05). Results are representative of the mean of 3 experiments depicted from Table 2.

Considering the cytogenetic diagnosis of the patients referred in Table 2, patients FA9 and FA10 were classified as mosaics and FA11 as a post-transplant chimera. Thus, they have a higher frequency of normal cells. What we can see is that, for these patients, the number of breaks per cell decreased to normal values with α-LA plus NAC treatment, which translates in an even greater reduction in the number of breaks per cell, of about 80%, *P* < 0.001 (Figure 3).

#### Effect of α-lipoic acid plus N-acetylcysteine on cell cycle progression in cultured lymphocytes from FA patients

**Figure 4:**
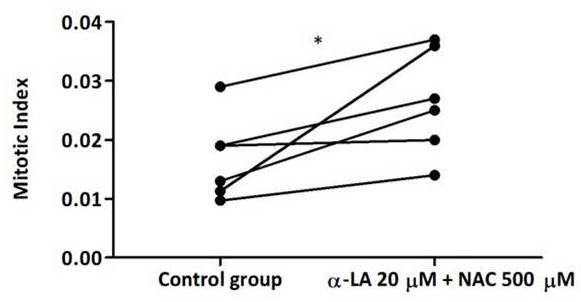
**Effect of α-LA + NAC on mitotic index of cultured lymphocytes from FA patients.** Lymphocyte cultures were treated with 20 μM α-LA plus 500 μM NAC. Mitotic index was evaluated as described in “methods” and the results are presented as absolute frequencies depicted from Table 3 (*P < 0.05).

MI was measured in lymphocyte cultures submitted to the three types of antioxidant treatments. The results displayed in Table 3 show that, in average, the α-LA treatment increased the MI from 0.17 (in control group) to 0.18, while NAC treatment increased to 0.19, and, most importantly, the combined use of both antioxidants resulted in a significant increase to 0.27, which clearly demonstrates that α-LA plus NAC treatment significantly increased the MI of cultured lymphocytes, *P* < 0.05 (Figure 4).

**Table 3:** Determination of mitotic index in cultured lymphocytes from FA patients

Mitotic index				
Patients	Control group	α-LA 20 μM	NAC 500 μM	α-LA 20 μM + NAC 500 μM
FA8	0,013	0,019	0,023	0,025
FA9	0,029	0,028	0,033	0,037
FA10	0,019	0,019	0,009	0,027
FA11	0,009	0,006	0,013	0,014
FA13	0,011	0,019	0,026	0,036
FA14	0,019	0,018	0,012	0,020
Mean ± SD	0.017 ± 0.007	0.018 ± 0.007	0.019 ± 0.009	0.026 ± 0.009

In control group no antioxidant treatment was done. Abbreviations are explained in Table 1. Paired t-test: Control group vs α-LA 20 μM + NAC 500 μM *P* < 0.05.

### Effect of α-lipoic acid, N-acetylcysteine and α-lipoic acid plus N-acetylcysteine on DEB- induced CI in lymphocyte cultures from FA patients

**Figure 5:**
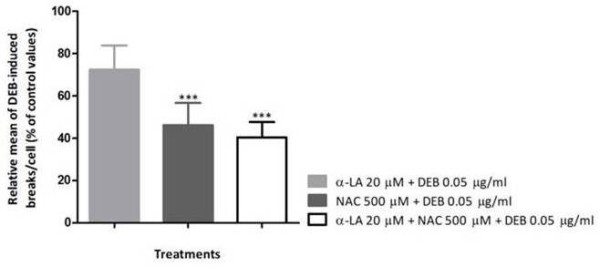
**Effect of α-LA, NAC and α-LA + NAC on chromosome breaks in DEB-induced cultured lymphocytes from FA patients.** Lymphocyte cultures were pre-treated with 20 μM α-LA, 500 μM NAC, and both antioxidants simultaneously at the same concentrations, 1.5 h before exposure to DEB 0.05 μg/ml for 48 h. The results are presented as the relative mean of breaks per cell (percentage of control values). Comparison of the relative mean of breaks/cell was made between control group and each antioxidant treatment (*** *P* < 0.001) and between treatments (*P* > 0.05). Results are representative of the mean of 14 experiments depicted from Table 4.

As shown in Table 4, lymphocyte cultures from FA patients pre-treated with α-LA, NAC, and α-LA plus NAC, and after exposure to DEB, showed a significant reduction in the number of breaks per cell compared to the control group (*P* < 0.05, *P* < 0.05 and *P* < 0.05, respectively). Comparing α-LA *vs* NAC treatments (*P* > 0.05), α-LA *vs* α-LA plus NAC treatments (*P* < 0.05) and NAC *vs* α-LA plus NAC treatments (*P* > 0.05) no differences were found in the reduction in the number of breaks per cell. Globally, the percentage of reduction in lymphocyte cultures pre-treated with α-LA was 28% (*P* > 0.05), with NAC was 54% (*P* < 0.001) and with α-LA plus NAC was 60% (*P* < 0.001), as shown in Figure 5. Once more, two distinct groups can be considered, a group of responders and another one of non-responders. In a total of 12 α-LA pre-treated lymphocyte cultures, 67% were responders, while in a total of 7 NAC pre-treated lymphocyte cultures, 86% were responders. In α-LA plus NAC pre-treated cultures 100% were responders. In a culture from one patient belonging to this last group, the number of breaks per cell decreased to values considered normal (below 0.1).

**Table 4:** Number of DEB-induced breaks per cell in cultured lymphocytes from FA patients

Mean number of breaks per cell							
Patients	Control group *(n)*	α-LA 20 μM *(n)*	% reduction	NAC 500 μM *(n)*	% reduction	α-LA 20 μM + NAC 500 μM *(n)*	% reduction
FA1	4.36 *(100)*	1.16 *(100)*	73.39	_	_	_	_
FA2	1.85 *(100)*	1.99 *(100)*	0.00	_	_	_	_
FA3	1.80 *(100)*	1.50 *(100)*	16.67	_	_	_	_
FA4	0.78 *(100)*	0.57 *(100)*	26.92	_	_	_	_
FA6	1.00 *(80)*	1.16 *(56)*	0.00	_	_	_	_
FA7	7.20 *(25)*	5.50 *(22)*	23.61	_	_	_	_
FA8	1.74 *(50)*	2.10 *(50)*	0.00	1.16 *(50)*	33.33	0.70 *(50)*	59.77
FA9	0.60 *(50)*	0.30 *(50)*	50.00	0.22 *(50)*	63.33	0.30 *(50)*	50.00
FA10	1.32 *(50)*	1.60 *(50)*	0.00	1.60 *(50)*	0.00	_	_
FA11	0.40 *(100)*	0.10 *(50)*	75.00	0.14 *(44)*	65.00	0.24 *(45)*	40.00
FA12	3.76 *(50)*	_	_	_	_	1.48 *(50)*	60.64
FA13	5.06 *(50)*	_	_	0.20 *(50)*	96.05	2.26 *(50)*	55.34
FA14	2.26 *(50)*	1.46 *(37)*	35.40	1.28 *(50)*	43.36	1.10 *(50)*	51.33
FA15	4.31 *(100)*	0.14 *(50)*	96.75	0.14 *(50)*	96.75	0.02 *(50)*	99.54
Mean ± SD	2.603 ± 2.019	1.465 ± 1.444		0.677 ± 0.641		0.871 ± 0.799	

In control group no antioxidant treatment was done and lymphocytes were exposed to DEB 0.05 μg/ml. The effect of the antioxidants was calculated by the percentage of reduction of the number of breaks per cell in the α-LA, NAC and α-LA plus NAC treatments relatively to control group. ― Unavailable data. Abbreviations are explained in Table 1. Paired t-test: Control group vs α-LA 20 μM *P* < 0.05; Control group vs NAC 500 μM *P* < 0.05; Control group vs α-LA 20 μM + NAC 500 μM *P* < 0.05.

## Discussion

In the present work we evaluated the protective effect of α-LA and NAC and, more importantly, the protective effect of a cocktail with these two compounds, against spontaneous and DEB-induced CI in lymphocyte cultures from FA patients. The obtained results provide an important and novel finding: it was clearly evident, for the first time, that the concomitant exposure to α-LA and NAC can drastically improve the genetic stability in lymphocytes from FA patients, *in vitro.*

Various reports have previously shown mitochondrial dysfunctions in FA cells, including distorted mitochondrial structures and peroxyredoxin 3 deregulation in FA-A, FA-C and FA-G cells[18] and mitochondrial matrix densification in FA-A fibroblasts after 8-methoxypsolaren photoreaction or UVA irradiation [17]. It is known that mitochondrial dysfunctions ultimately lead to a defect in energy transduction, with increased formation of ROS, which, by itself, can lead to lower cellular energy charge, oxidative modification of DNA, protein and lipids. In addition, there is also a possibility of a positive feedback cycle between ROS formation and mitochondrial damage, exacerbating the processes of cellular dysfunction (reviewed in Tarnopolsky) [26]. It was already demonstrated that GSH protects mitochondrial DNA from oxidative damage in human lymphocytes and that GSH depletion increases their susceptibility to mitochondrial DNA damage [36]. It is noteworthy that GSH levels are downregulated in FA patients [37]. Therefore, it may be postulated that such imbalance in the redox state of FA cells can be counteracted by antioxidants, in particular mitochondrial nutrients, like α-LA, and other antioxidants that can replace or replenish GSH levels, like NAC, as it was demonstrated in the present study.

Hypersensitivity of lymphocytes to the clastogenic effect of DEB is a common biomarker of FA. Previous studies have shown that DEB acts as a bifunctional alkylating agent, exhibiting both inter-strand and intra-strand DNA cross-linking ability [38] and that is redox-active, generating ROS that can damage DNA [8]. Furthermore, acute exposure of normal human lymphocytes to DEB is associated with GSH and ATP depletion [10], which indicates that DEB can also cause mitochondrial dysfunction. The exacerbation of OS and DNA cross-linking in FA lymphocytes, through DEB-mediated effects, can reproduce damaging conditions present in severe clinical FA situations, which theoretically extends the applicability of the present results from bench to bedside.

**Figure 6:**
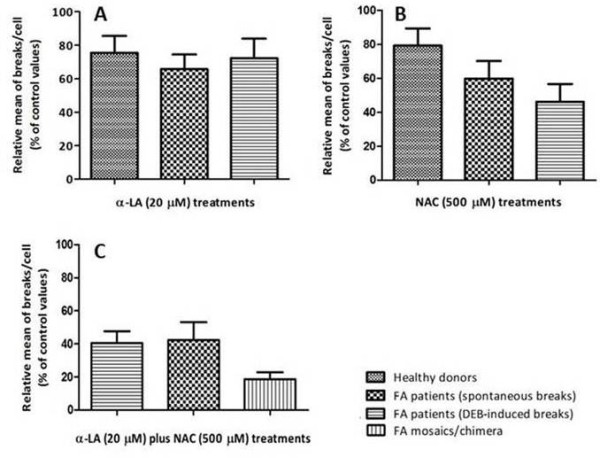
**Comparison between the different studied groups according to the antioxidant treatments.** Comparative analysis of the effect of α-LA (**A**), NAC (**B**) and α-LA plus NAC treatments was performed between lymphocyte cultures from the four studied groups: healthy donors, FA patients (spontaneous breaks), FA patients (DEB-induced breaks) and FA mosaic/chimera patients. All values were depicted from Figures 2, 3 and 5.

Under the present experimental conditions, the α-LA effect, measured by the reduction in the number of breaks per cell, was similar (around 30%) in the three studied groups: DEB-induced lymphocyte cultures from HD and spontaneous and DEB-induced cultures from FA patients (Figure 6A). This indicates that α-LA effect was independent of DEB exposure or health condition of the studied subject. On the other hand, we verified that NAC effect in these same three groups seems to be dependent of the treatment and health condition (Figure 6B), since the reduction in the number of breaks per cell was greater in DEB-induced lymphocytes from FA patients. These results are in agreement with previous studies, showing that NAC inhibits ROS production and apoptosis induced by DEB in human lymphoblasts [8] and that NAC protects human lymphocytes from acute *in vitro* exposure to DEB [10]. Last but not least, we also verified that the effect of the combined use of α-LA plus NAC is independent of DEB exposure, as shown in Figure 6C, with a 60% of reduction in the number of breaks per cell in both groups of FA patients, and even more effective in cultures from FA patients with higher frequency of normal cells, such as FA mosaics or FA chimera, in which the reduction was of about 80%. Furthermore, the measurement of spontaneous chromosomal breaks was of particular importance, as it rules out the possibility that the main anti-chromosomal break effect of α-LA and NAC is due to a direct interaction of these agents with DEB.

Additionally, MI was measured in lymphocyte cultures from FA patients submitted to all antioxidant treatments. The reason for this assay was to evaluate if the decrease in CI was due to an increase in mitotic proliferation, with a consequent shortening of the cell cycle arrest in G2, as it was demonstrated for α-tocopherol by Pincheira et al [23]. What we observed is that MI increased in all antioxidant treatments, especially with α-LA plus NAC treatment, suggesting a relation between a decrease in CI and a shortening of cell arrest in G2. We cannot exclude, however, that, in lymphocyte cultures from mosaic and chimera patients, the increase in MI can be due to a positive selection of normal cells, with a consequent increase in apoptosis.

The results now obtained suggest that α-LA plus NAC cocktail may be useful to keep chromosome stability in FA patients, which can be very important to block or delay the progression of the disease. This cocktail may be even more effective when applied to FA mosaics and FA chimeras, after bone marrow transplant, as we observed *in vitro*. It must be stressed that bone marrow transplant only resolves bone marrow failure, curing the hematological manifestations. However, CI is not mitigated, and predisposition to cancer, other than leukemia, persists, especially solid tumors [3,39,40]. Thus, controlling CI in FA patients after bone marrow transplant could be very important.

Additionally to increasing chromosomal stability, α-LA has also important cellular roles in the regulation of gene transcription, inhibition of the activation of NF-kB (nuclear factor kappa-light-chain-enhancer of activated B cells) and activating protein 1, [41,42] while NAC apparently modulates cytokine concentrations, as interleukin(IL)-1, TNF-α (tumor necrosis factor-alpha) and IFN-γ (interferon-gamma) [30]. These pharmacological effects may further contribute for the therapeutic usefulness of α-LA plus NAC cocktail by avoiding the inflammatory process, mostly frequent in FA children patients [43,44]. Of note, some studies concerning the immunological response of FA patients demonstrated lymphocyte dysfunction and high levels of TNF-α [45]. Low production of IL including IL1, IL2, and IL6, as well as IFN-γ and granulocyte-macrophage colony stimulating factor have also been reported [46].

The present work might open a novel strategy to deal with FA disease. The choice of these antioxidants, apart from their pharmacodynamics, takes into account that both α-LA and NAC have been already approved for safe human use with minimal or no adverse effect, showing an excellent safety profile. NAC has been in clinical use for more than 30 years, in conditions characterized by depletion of GSH or OS, as cancer, AIDS, pulmonary diseases, heart diseases, as well as in acetaminophen overdose [30,47,48]. Thus, in conditions characterized by a chronic excessive OS and in clinical situations where GSH levels are decreased, NAC appears to be a highly effective drug, and may be also recommendable in the prevention/treatment of FA. α-LA has been used, especially in Germany, for over 30 years, in the treatment of diabetic polyneuropathies. Several evidences support a role for α-LA as a mitigator of OS in type 2 diabetes [49], this drug being also used in the treatment of atherosclerosis, cardiovascular diseases, cataracts, neurodegenerative diseases, liver diseases and AIDS [50]. Besides the well-known characteristics as antioxidant and as mitochondrial nutrient, α-LA can be administrated orally, since it is easily absorbed in the stomach, it crosses blood–brain barrier, due to its amphipathic properties, and is not toxic at doses used for prophylactic and therapeutic purposes [50]. Furthermore, α-LA and NAC are also inexpensive drugs, which is a great advantage compared with other proposed therapies, namely gene therapy [51], and others that are now available, such as androgen therapy [52], allowing an easy access by all FA patients. Therefore, this cocktail may be immediately tested in phase III clinical trials for treating chromosome instability. Moreover, a FA prevention trial could also be designed to see whether the administration of these drugs can prevent the development of leukemia, in an initial phase, and solid tumors in an advanced form of the disease. The proposed clinical trial, using the combined administration of α-LA and NAC also poses a few challenges. FA is a rare disease, which implies a small population of patients, most of them children or teenagers. This requires a strong adhesion to the new approach by parents and family. In addition, the efficacy may vary among FA patients, as we observed by the presence of responders and non responders. However, this challenge may be easily overcome by studying the response of each individual in an *in vitro* assay before starting the therapy.

A large number of epidemiological studies have shown health improvements in association with the consumption of dietary antioxidants as part of food. Several studies have shown that the provision of individual antioxidants as supplements are either ineffective [53], or can be deleterious to health [54]. Part of the reason that large doses of exogenous single antioxidants can be deleterious is that every antioxidant can also function as a pro-oxidant and antioxidants function optimally when several are present and can function as redox couples [26]. This fact may explain the success of our cocktail, α-LA plus NAC. They act as an effective redox couple, and in two distinct but closely related processes: α-LA acts as a mitochondrial nutrient and NAC as a GSH precursor and direct ROS scavenger.

## Conclusions

The present study provides an important and novel finding that may have immediate clinical applicability: it was clearly evident, for the first time, that the concomitant exposure to α-LA and NAC can drastically improve the genetic stability in lymphocytes from FA patients*.* Since the available information points to an association of cellular phenotype and clinical features with the occurrence of both cancer-proneness and OS in FA disorder, these results suggest that α-LA plus NAC can be an effective antioxidant prophylactic cocktail to be applied, *in vivo*, in FA patients, with a consequent block or delay in their characteristic bone marrow failure and early cancer development. On the pre-clinical perspective, mechanistic studies are still required to understand why these antioxidants together are so effective in the reduction of CI in lymphocytes from FA patients, providing, this way, further clues to know what is falling in FA cells.

## Abbreviations

8-OHdG, 8-hydroxy-2’-deoxyguanosine; 8-oxoG, 7,8-dihydro-8-hydroxyguanine; AIDS, Acquired immune deficiency syndrome; CI, Chromosome instability; DEB, Diepoxybutane; DMSO, Dimetilsulfoxide; FA, Fanconi anemia; GSH, Reduced glutathione; HD, Healthy donor; IL, Interleukin; INF-γ, Interferon-gamma; MI, Mitotic index; NAC, N-acetylcysteine; NF-kB, Nuclear factor kappa-light-chain-enhancer of activated B cells; OS, Oxidative stress; PRDX3, Peroxiredoxin 3; ROS, Reactive oxygen species; TNF-α, Tumor necrosis factor alpha; α-LA, α-lipoic acid.

## Competing interests

The authors declare no competing financial interest.

## Authors’ contributions

FP designed experiments, performed research, analyzed data and drafted the manuscript. RS performed research and analyzed data. APF, CG and JB provided blood samples and clinical data from FA patients, analyzed the experimental data. BP and FC coordinated the study, designed experiments, analyzed data, critically revised the manuscript and gave final approval of the version to be published. All authors read and approved the final manuscript.
